# Full-field flicker evoked changes in parafoveal retinal blood flow

**DOI:** 10.1038/s41598-020-73032-0

**Published:** 2020-09-29

**Authors:** Raymond L. Warner, Alberto de Castro, Lucie Sawides, Tom Gast, Kaitlyn Sapoznik, Ting Luo, Stephen A. Burns

**Affiliations:** School of Optometry, Indiana University, Bloomington, 47405 USA

**Keywords:** Retina, Optics and photonics

## Abstract

When retinal activity is increased by exposure to dynamic visual stimuli, blood vessels dilate and the flow of blood within vessels increases to meet the oxygen and glucose demands of the neurons. This relationship is termed ‘neurovascular coupling’ and it is critical for regulating control of the human retinal vasculature. In this study, we used a recently developed technique based on a dual-beam adaptive optics scanning laser ophthalmoscope to measure changes in red blood cell velocities, vessel diameter, and flow in interconnected small parafoveal retinal vessels (< 50 µm) of nine healthy participants. A full-field flicker stimulus was presented onto the retina to induce a vascular response to neural activity. Flicker stimulation increased blood velocity, vessel diameter, and therefore flow in arterioles, capillaries, and venules in all nine subjects. ANOVA and post hoc *t*-test showed significant increases in velocity and flow in arterioles and venules. These measurements indicate that the mechanism of neurovascular coupling systematically affects the vascular response in small retinal vessels in order to maintain hemodynamic regulation in the retina when exposed to visual stimulation, in our case flicker. Our findings may provide insight into future investigations on the impairments of neurovascular coupling from vascular diseases such as diabetic mellitus.

## Introduction

The body must deliver more blood to active neural regions in order to meet their increased metabolic demand^1^. Increases in demand from neural activity results in dilation of feeding blood vessels in order to maintain oxygen and glucose concentrations^2^. This vascular response is termed neurovascular coupling and it is essential to maintaining healthy hemodynamic function^3,4^. Studies have shown increases in blood flow in the primate retina using techniques such as labeled microspheres^5^ and laser targeted angiography^6^. Similarly cats also showed increases in blood flow response at the optic nerve head when exposed to a 30° luminance flicker stimulation using a laser Doppler fundus camera^2^. These studies showed variations in response depending on the stimulus conditions, consistent with what is known of retinal visual processing^7^. In rodents similar results are found with flicker-evoked increases in flow in both mice, using optical coherence tomography angiography^8,9^ and rats, using fluorescein angiography^10^. For humans neurovascular coupling has been intensively investigated in the brain^11,12^, where it is the source of the BOLD signal that underlies fMRI^13^ as well as in the retina using noninvasive imaging.

For better understanding of neurovascular coupling in small vessels the human retina is especially useful^14,15^ for two several reasons. First, it is a portion of the central nervous system that scatters light minimally and is thus uniquely accessible to optical measurements. Second the vasculature of the retina is arranged in multiple layers^16^ with relatively few connections that carry blood axially from inner to outer layers or vice versa. This laminar organization of the retinal vasculature allows optical modalities to capture flow at vessel sizes down to capillaries using two-dimensional imaging, without the need to track cells in 3D and finally humans can be measured noninvasively without anesthesia.

To date, numerous techniques have been used to measure blood flow in the human retina, including laser Doppler velocimetry^17^, laser Doppler flowmetry^18^, Doppler optical coherence tomography^19^, the retinal vessel analyzer^20^, fluorescein angiography^21^ and scanning laser ophthalmoscopy^22–25^. Although these techniques acquire flow measurements with high signal-to-noise ratio, there are limitations. Most of these techniques do not work well for smaller retinal vessels and thus are best suited to providing a more global measure of neurovascular coupling in the retina and do not allow the study of neurovascular coupling within local regions of the retina. More recently techniques have been developed based on measuring blood flow using adaptive optics imaging that allow tracking leukocytes^26,27^ as well as red blood cells^22,24^.

As retinal imaging techniques measure blood flow increases in response to flicker stimuli^14,19,28^, the measurement of blood flow responses in small retinal vessels in humans is not fully understood. We recently developed a new imaging technique which can measure red blood cell velocity in small vessels, including capillaries^29^. This combines the high resolution provided by our adaptive optics scanning laser ophthalmoscope (AOSLO), together with the high temporal resolution provided by dual offset imaging, to measure systematic changes in velocity, diameter, and flow in small retinal vessels^30^. In the current paper we investigate the ability of this dual-beam imaging approach^29^, to measure activity induced changes in red blood cell (RBC) velocity in small arterioles, venules, and capillaries (< 50 µm) as well as changes in vessel diameter, and in RBC flow using a full-field flicker stimulus under three conditions: before flicker stimulation, during flicker stimulation, and after flicker stimulation.

## Results

### RBC velocity response to flicker stimulus

**Figure 1:**
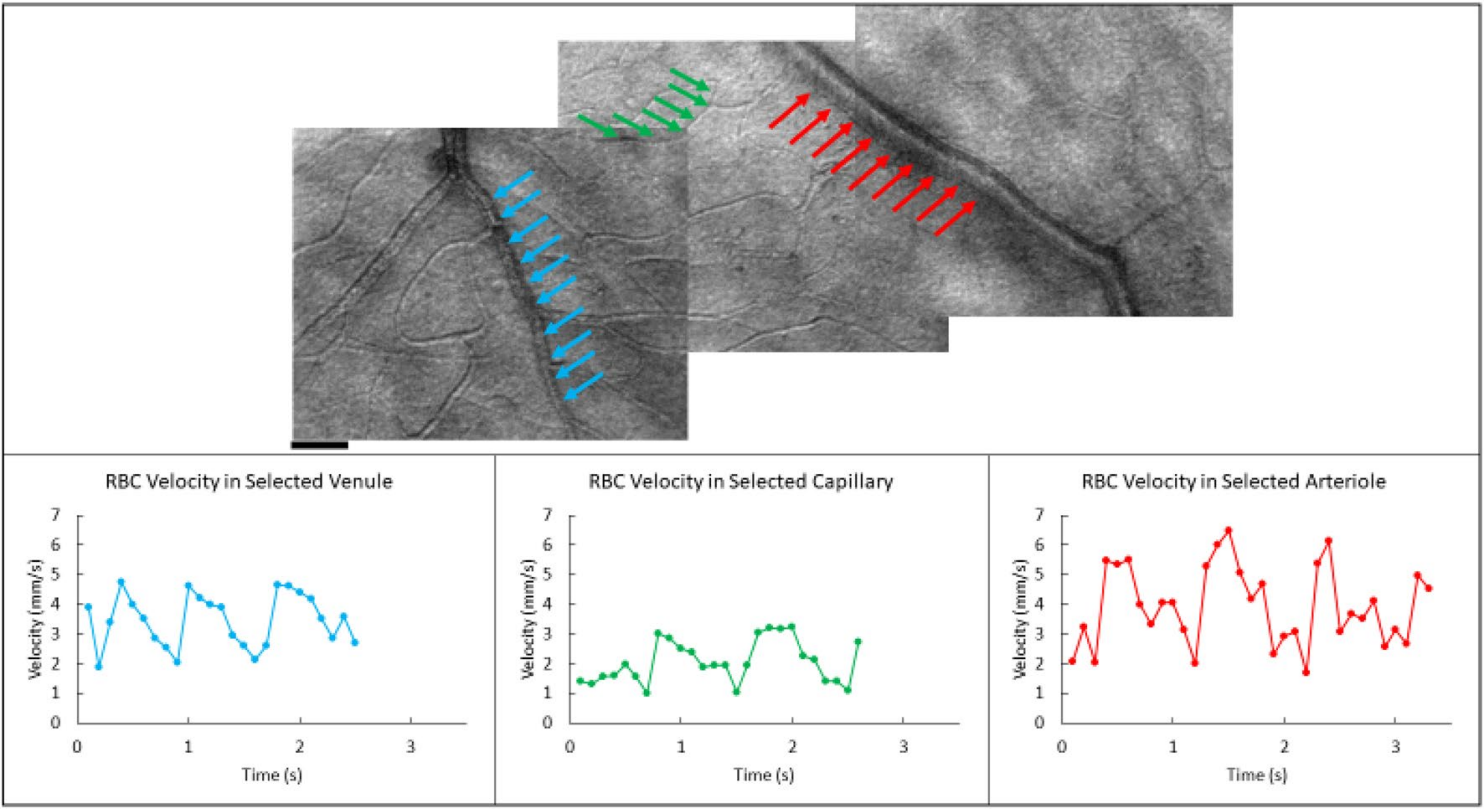
A montage of a region of interconnected vessels in the parafoveal retina in one subject and the respective velocity plots of the average RBC’s over a three second video. Venules and arterioles were selected due to both the size of each vessel (< 50 µm) and whether a capillary region between both vessels are visualized for analysis. The video for each of the selected vessels were taken at different times and therefore the pulsatility will vary over time between each vessel. Scale bar is 50 µm.

The velocity of RBCs was measured within an interconnected vascular network in the superficial capillary plexus consisting of an individual arteriole, capillary, and venule in nine subjects (as shown in Fig. 1). Three second videos of the individual vessel were recorded for 2 min for each condition and RBC velocities were averaged. Average velocities at baseline ranged from 2.07 mm/s to 3.79 mm/s for arterioles, 0.73 mm/s to 2.52 mm/s for capillaries, and from 2.05 mm/s to 3.63 mm/s for venules. The change in velocity was normalized by dividing by the average velocity measured in the baseline condition for each blood vessel. For the duration of the full-field flicker stimulus, the average RBC velocity increased by 10% for arterioles (± 2% standard error of the mean), 15% for capillaries (± 7% standard error of the mean), and 12% for venules (± 2% standard error of the mean). After flicker offset, velocities for the arterioles and venules returned to baseline values. Capillaries did not always return to baseline, showing a 6% increase in velocity relative to baseline. A single-factor ANOVA showed that the average velocities under the Full-Field Flicker stimulus significantly varied between the different conditions for the arterioles [F (2, 24) = 15.87, p < 0.001], and venules [F (2, 24) = 10.99, p < 0.001]. A post hoc *t*-test confirmed that average velocities increased during flicker stimulus condition (*p* < 0.001; one-tailed *t*-test). The increase in capillaries velocity was not significant [F (2, 24) = 2.49, p = 0.10] in part due to the high variability in capillary velocities (supplementary Fig. 1).

The coefficient of variation (C.V.) was used to measure fluctuations in RBC velocity in each type of vessel for each experimental condition. Baseline variability in RBC velocity in arterioles decreased from 13 to 11% during flicker stimulation, then back to 13% after stimulation. The variability of capillary velocity decreased from baseline 19% to 17% during flicker stimulus, then back to 19% after flicker stimulation. Variability in venules decreased from 12 to 9% during flicker stimulus, then increased to 11% after flicker stimulus.

### Vascular diameter response to flicker stimulus

**Figure 2:**
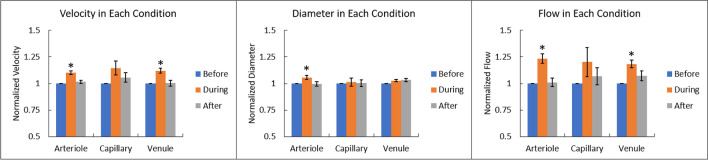
Bar plot of the change of velocity, diameter, and flow for each with respect to the baseline condition among all subjects. Error bars indicate standard error of means for each measurement.

To compare changes in the inner diameter of the measured blood vessels as a result of flicker stimulation, measurements for each arteriole, capillary, and venule were normalized by dividing the diameter in each stimulus period by the diameter at baseline. Diameters at baseline ranged from 13.66 µm to 32.09 µm in arterioles, 6.01 µm to 9.17 µm in capillaries, and 17.22 µm to 41.24 µm in venules. During flicker, the diameter increased 6% in arterioles (± 2% standard error of the mean), 3% in venules (± 1% standard error of the mean), and 1% in capillaries (± 4% standard error of the mean) (Fig. 2). A single-factor ANOVA found statistically significant differences in diameter for arterioles [F (2, 24) = 4.81, p = 0.017], but not for capillaries [F (2, 24) = 0.074, p = 0.93], or venules [F (2, 24) = 2.39, p = 0.11]. Individual diameters between subjects varied in response as shown in supplementary Fig. 2.

### Total flow response

RBC flow was normalized relative to baseline conditions for each vessel. Average flow at baseline ranged from 0.02 µl/min to 0.10 µl/min in arterioles, 0.002 µl/min to 0.007 µl/min in capillaries, and 0.03 µl/min to 0.20 µl/min in venules. Flow in arterioles significantly increased relative to baseline by 23% among all subjects during flicker stimulation (± 4% standard error of the mean), then decreased 22% during the recovery period [F (2, 24) = 14.82, p < 0.001]. Flow in venules significantly increased 18% during flicker stimulation (± 4% standard error of the mean) and then showed a 11% decrease during the recovery period [F (2, 24) = 7.62, p < 0.001]. Post hoc t-test found significant increases in flow to occur during flicker stimulation (*p* < 0.001; one-tailed *t*-test). Although capillary flow trended toward an increase on average by 20% compared to baseline ($\pm$ 14% standard error of the mean) with a 13% decrease in flow after flicker stimulation, a single-factor ANOVA showed no significant increase in average flow as [F (2, 24) = 1.27, p = 0.103]. Figure 2 illustrates the normalized changes in velocity, diameter, and flow for arteriole, venule, and capillary vessel among all subjects during each condition. Individual subjects velocity, diameter, and flow changes during each condition are provided in the Supplementary figures.

**Figure 3:**
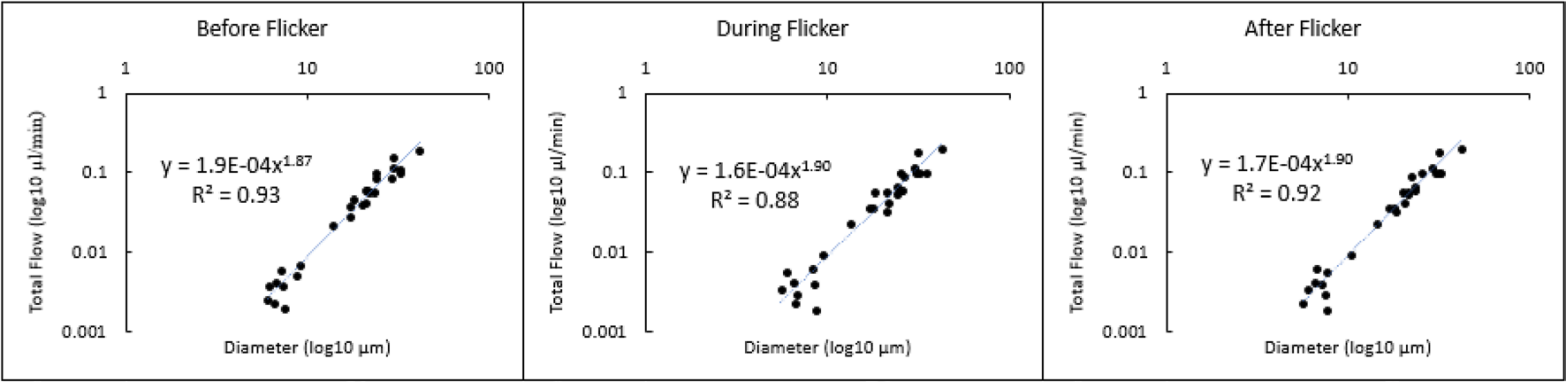
Average total flow for all vessels measured over the corresponding inner diameter: before (left), during (middle) and after (right) flicker stimulation.

Increases in total flow for each experimental condition are shown in Fig. 3 plotted as a log/log function of vessel diameter. Data were well fit by a power law with an exponent of 1.87 during baseline condition with the exponential fit accounting for 93% of the variance. During the full-field flicker condition the average flow computed across the vessel sizes in the flicker condition produced an exponent of 1.90 with an r^2^ of 0.88. Flow during the post-flicker duration were best fit with an exponent of 1.90 with a correlation coefficient of 0.92. None of these differences were significantly different.

## Discussion

When the retina is exposed to flicker, blood flow is increased in order to sustain metabolic demand driven by neighboring neurons^2,31^. This increase in neural activity causes neighboring blood vessels to dilate and supply additional oxygen and nutrients. In this study, we have shown that introducing a flicker stimulus onto the retina will upregulate flow in local, interconnected blood vessels in the inner retina as a result of increased metabolic demand. With the dual-beam AOSLO, we have the resolution to measure systematic changes in the velocity, diameter and ultimately flow in small retinal vessels. While techniques such as SLO fluorescein angiography and leukocyte visualization^6^ have been used to show changes in capillary flow, these are more difficult to use. For example, fluorescein angiography requires injection of a dye to visualize cells in the vasculature, as well as other AO techniques^24^. These techniques have often used visible light, and as a result there is a strong neural response to the imaging light itself. We used near infrared light, which while it was still visible and because imaging was performed by raster scanning, it could generate a blood flow response to the imaging light. To minimize this interaction between imaging and response we chose the average luminance of our visual stimulus based on results of pilot experiments. In the pilot experiments we varied the luminance of the visual stimulus. A brighter time average luminance would shift the adaptation state of the retina, decreasing the neural response to the imaging field which is flickering at 29 Hz. We chose the final luminance to be as low as possible, to avoid bleaching and ganglion cell saturation, while also increasing the size of the stimulus induced increase in blood flow. The pilot experiments also suggest that the background decreased the variability in the blood flow response across participants.

Our measures of velocity and flow with the new dual offset imaging system are similar to previous measurements for vessels with similar sizes^21,22,24,29^. Although flow increases with visual stimulation in interconnected parafoveal vessels ranged from 18–23%, the vascular response is relatively small compared to studies measuring flow changes in larger retinal vessels under similar conditions which found increases ranging from 34% up to 66%^32–34^. This could be a function of the difference in the physical conditions for large and small blood vessels. In vessels > 100 µm, devices such as the retinal vessel analyzer measure changes in diameter and then assumed that the flow dynamics reflect that of a Newtonian fluid and exhibit Poiseuille’s law, where velocities at the vessel wall is near 0 and velocity distribution across the vessel is parabolic^22^. Because of this, small changes to the diameter of the vessel can result in significant changes in flow as shown by Poiseuille’s law.

Flow upregulation in response to flicker stimulation was found in arterioles and venules in all subjects, however flow in capillaries only increased in 7 out of the 9 vessels measured. Because of the physical constraints that flow into a region must equal flow out of the region, this result is perplexing. However, capillary flow varies can be attributed to a number of factors including both physiological and methodological causes. Capillaries have pericytes that intermittently wrap around the vessel that help regulate changes in the diameter^35^. Although showing similar characteristics to that of the smooth muscle cells in arterioles, the constriction and dilation mechanisms for capillaries are not fully understood^36^ and are inherently more variable on an individual basis^37^, an effect we have informally observed in our own measurements. We measured only a single capillary in the selected region sub-served by multiple capillaries for each subject. This was done due to the high stability and signal to noise ratio needed to resolve blood cells within the same capillaries over the complete imaging session. Because maintaining imaging on the same capillaries over the entire time course of the experiment is difficult (see below), we have chosen an individual capillary that shows detectable RBCs over the course of the experiment. Because of this, the total flow within the capillary network may not exactly represent the average response of individual capillaries^36^. There are also changes in the timing between capillary layers in cats, and this may be impacting these measurements in the superficial capillary plexus^10^. Even though not all capillaries returned to baseline within the 2-min measurement period, some did, and it is likely that longer measurement periods, although difficult for human subjects, would have seen capillaries flow return to baseline levels. Arterioles contribute significantly to the temporal and spatial changes in capillary perfusion, where arteriole velocities return to baseline, the change in perfusion pressure will also cause changes in flow at the capillary level^38^. The return to baseline is also impacted by the overall variability in capillary blood flow. When comparing the C.V. of RBC velocity in each vessel for each imaging condition, we find that capillaries on average have the largest variability within a given imaging condition. It is important to note that velocities are very slow in the capillaries, and thus displacements are on the order of a few microns, and while we resample the data prior to averaging to allow more accurate centroiding of the locations, and thus the displacement, there is still room for error. However, we do not believe that measurement error is the most likely factor explaining this variation in velocity. Rather, we believe it is an intrinsic variability in capillary velocity^37^. The small arterioles and venules we measured were more stable in their velocity than capillaries, presumably because they integrate flow over a number of capillaries. Future investigations measuring multiple capillary velocities simultaneously may give insight into the total flow dynamics over a region of capillaries and elucidate how flow is regulated into and out of a given capillary bed.

We measured vessels dilations between 1 and 6% between capillaries, venules, and arterioles during flicker stimulation which is in range of the 2–5% dilations measured in other studies^17,20^. The amplitude of the diameter increase as a result of flicker stimulation is smaller than dilations found in larger vessels. To increase total flow by (for instance) 10% in a large vessel requires a smaller proportional change in diameter than for a smaller vessel due to the accelerating relation between size and flow (Eq. 1). Arterioles showed the largest amplitude of dilation with 6% increases during the full-field flicker stimulus, venules having 3% dilation in response to flicker, and capillaries with 1% dilation. A possible reason as to why arterioles exhibit the largest changes in diameter may stem from the smooth muscle cells that wrap along the vessel and allow vessels to dilate and constrict accordingly. These dilations are similar to the results of Nagel and Vilser who found retinal arteriole dilations up to 7.2% ± 2.4%^39^. Overall, the amount of vessel dilation contributing to overall flow output ranges from 4 to 14%, consistent with previous findings with similar and larger vessels^15,19,32,40^. Although not mentioned in the final results, we found no significant differences in diameter changes during flicker stimulation with respect to vessel size, where arterioles, venules, and capillaries of differing sizes exhibited different ranges of dilation/constriction during flicker stimulation.

Murray’s law posits that the energy needed to maintain vascular function and flow is optimized^41^. That is, the retinal vascular tree minimizes the amount of energy required to maintain blood flow to a region. We find that that the relation between diameter and flow does not show the power of three expected from Murray’s law, a result that is consistent with previous measurements of small retinal vessels^17,42^. The exponents relating flow to inner diameter in this study lie between the exponents found for vessel branching ratios, which ranges from 1.69–2.10 in branching vessels less than 50 µm^42^. Other velocity measurements, such as those using targeted dye delivery, found vessels between 20 µm to 80 µm to exhibit power law exponents of 2.90, less than that predicted by Murray’s law though larger than the values found here^21^. AOSLO measurements for larger vessels using a different technique also found an exponent closer to 3^43^. We do expect deviations from Murray’s law for small vessels for a number of reasons. First, the Fåhræus–Lindqvist effect becomes important^44^. This effect arises from the separation of cells into a column with a cell free region, resulting in a consequent decrease in the viscosity of the blood. This flow of RBCs in small vessels forces plasma along the edges of the inner walls of the vessels presumably requiring less work. Studies show that the magnitude of work deviating from Murray’s Law (that is total flow relative to vessel size) would be no greater than 1/6 of the optimized work (which in Murray’s Law is the power of 3)^45^. Our exponents are even lower than expected from the Fåhræus–Lindqvist effect, but there are several other considerations that come into play for retinal vessels. First, the wall to lumen ratio is considerably larger for these small vessels^46^ than for the slightly larger vessels used by Murray to derive Murray’s law, and this would increase the metabolic demand for maintaining the capillaries. Second, the retina is under strong pressure to minimize the impact of light scatter on retinal sensitivity, as evidenced by the presence of the foveal avascular zone. That is, the network itself may be deviating from ideal conditions in order to decrease the impact of scattered light. Finally, because the retinal vessels maintain a blood brain barrier, they actually have a larger metabolic demand, and therefore there may be selection pressure to increase the volume delivered for a given surface area of the vascular lumen. Whatever the actual reasons, at small scales the vascular system is deviating from Murray’s law as indicated by both the branching pattern^42^ and the flow^17^.

Although sufficient for measurements reported here, the current imaging approach has limitations. One is the size of the imaging field. Since it is a small imaging field of 1.3° × 1.1° (390 × 330 µm), it is difficult to quickly locate targeted regions of interest. We accounted for this by first creating a small montage and recording positions of the desired regions. However, with subjects who are not well-trained or accustomed to participating in experiments, maintaining eye positions, even with a fixation target can also be difficult. Eye motions cause the imaged regions to move during the experiment and because we wanted to capture several cardiac cycles to ensure an adequate estimate of the average velocity, we could only measure vessels that stayed within the imaging field. Voluntary or involuntary eye movements as well as other bodily movements may affect the image quality as well. Other eye movements such as drifts, microsaccades, or head motion can interfere with the recording of the image, although we have a fairly sophisticated post-processing approach to removing eye motions. To account for this, we collect over 7 min for each targeted vessel to collect at least 1 min of data for each individual. We include multiple breaks in our protocol as dry eye and fatigue can influence the AO correction during image acquisition and ultimately reduce the image quality. It has been reported that peak vascular response occurs 10–15 after seconds of flicker stimulation, so averaging over an entire minute’s worth of data is sufficient^2,47^.

The ability to measure local blood flow and its regulation may have important clinical implications. Since the vascular response to neural activity is essential for maintaining healthy vision^17,31^, disruptions to neurovascular coupling can induce severe vision loss. Studies utilizing flicker stimuli in patients with moderate non-proliferative diabetic retinopathy and glaucoma have shown diabetic individuals to have reduced flow rates and diminished responses to neural activity^48–50^. Ultimately, understanding the physiological mechanisms in normal control subjects will act as a guideline to healthy hemodynamic regulation in the retina.

## Methods

### AOSLO system

RBC velocities were measured using a new dual-beam AOSLO which has been previously described^29,51^. Briefly this system combines a Shack-Hartmann Wavefront Sensor with two deformable mirrors (Mirao 52e DM, Imagine Eyes, Orsay, France; Multi-DM, Boston Micromachines Corporation, Cambridge, MA) in a woofer tweeter configuration^52^.

**Figure 4:**
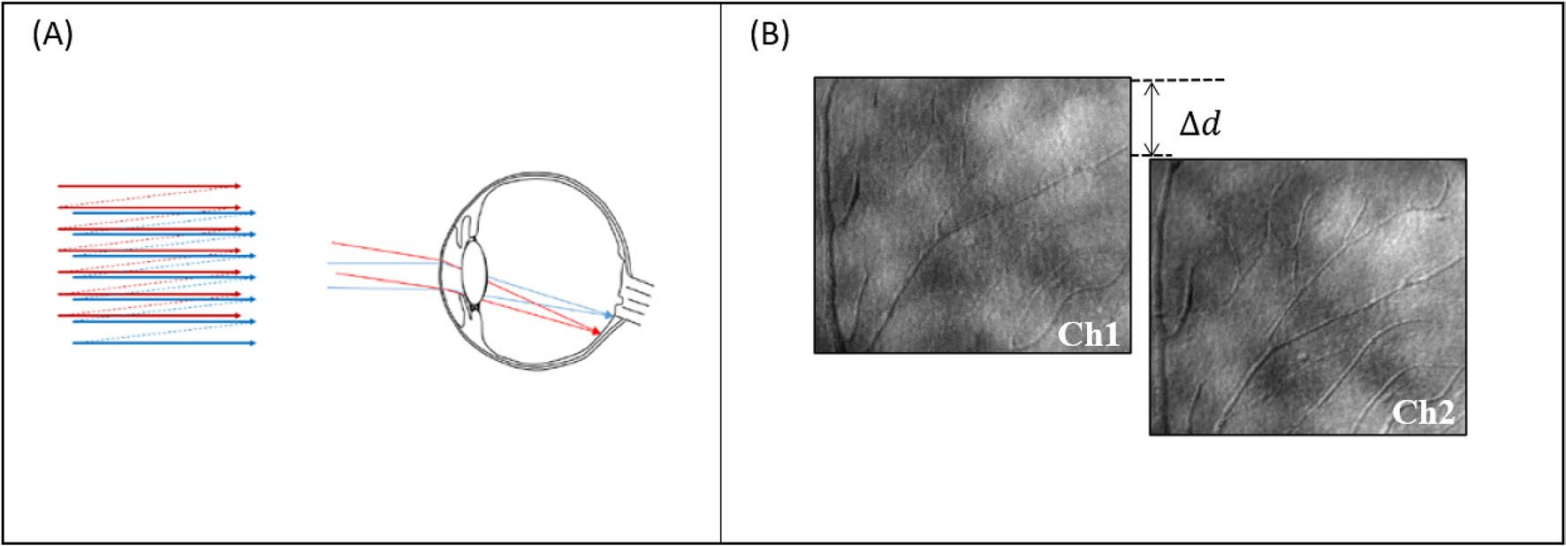
(**A**) An overview of light delivery with the dual-beam AOSLO. One beam is vertically displaced relative to the other allowing light to form at different areas at the retina. (**B**) Illustration of the concept of the dual offset imaging. Two images the same ROI a subject’s retina are acquired simultaneously, but with a spatial offset. The vertical displacement $(\Delta d)$ of the second channel allows us to image the same region of the retina, at two different times with a much smaller temporal separation than required to image a single frame.

Two imaging beams were simultaneously delivered (769 nm and 842 nm) from a supercontinuum laser (NKT Photonics, Birkerod, Denmark) and focused onto the retina. The two beams were scanned horizontally at a frequency of 15.1 kHz and vertically at a frequency of 29 Hz to image a 1.3° × 1.1° region of the retina. The light coming back from the eye is spectrally separated and delivered to two avalanche photodiodes, generating images of 780 × 520 pixels at frame rates of 29 fps. In order to achieve the temporal resolution necessary to measure velocities without aliasing, the angle between the two beams is controlled at a pupil conjugate plane to vertically displace one of the beams on the retina compared to the other beam. This configuration allows a common retinal area to be imaged at two moments in time, but each within the total frame time of the imaging system. In the current study, the two imaging beams were offset approximately 50 lines vertically to produce a temporal offset of 3.31 ms (50 lines * 15,100^−1^ s/line). Figure 4 illustrates the technique used to decrease the effective sampling interval of the raster scan displacement of the two imaging beams. To visualize individual red blood cells, multiply scattered light is collected with a 10 Airy Disk Diameter (ADD) aperture offset at least 6 ADD from the center of the Airy disc. The airy disk diameter for both of these imaging channels is 50 µm at the plane of the confocal pinhole. Light passing through these offset apertures is used to collect multiply scattered light images^53^. For a fully dilated pupil the resolution of the system is approximately 2 μm^52^. During imaging sessions, the experimenter is provided a plot of the location of the AOSLO imaging field superimposed on a 30 degree scanning laser ophthalmoscopic image (Heidelberg Spectralis, Heidelberg, Germany)^54^ of the subject’s fundus to easily navigate to vessels of interest.

### Full-field flicker stimulus

A visual stimulator was integrated into the AOSLO system. This stimulator provided a 25° × 11° region that was illuminated by light derived from a small digital light projector (DLP) (TI 4500, Texas Instruments, Dallas TX). The DLP was integrated into an optical relay system that included a Badal optometer for independent focusing of the visual stimuli. The visual stimulator was combined with the AOSLO beam just prior to entering the eye using a pellicle. A programmable, stable fixation stimulus was provided for the subject during all stimulus conditions. Luminance flicker was generated and delivered to the DLP using custom MATLAB software (Mathworks, Natick, MA) at a mean luminance of 3.7 lx. The full-field flicker stimulus used in this experiment was achromatic with a square-wave modulation of the entire field with a maximum illuminance of 6.6 lx and a minimum illuminance of 0.6 lx at 10 Hz. These illuminance intensities and modulation rates were similar to previous literature studying neurovascular coupling behavior in the retina in order to induce high metabolic activity in the retina^3,14^.

### Image acquisition and processing

Images were recorded as approximately three-second videos of 100 frames. Images from four video detectors were acquired simultaneously, although for this experiment we used data from only two detectors collecting multiply scattered light in similar directions with identical aperture positions. Image distortions resulting from the speed variations of the horizontal scanner, which moved at a sinusoidal velocity, were corrected prior to image registration. Frames were aligned and averaged via MATLAB and a template frame was automatically selected based on parameters such as brightness and cross-correlation values compared to previous frames using a strip alignment procedure^55^.

### Blood flow computations

RBC velocity measurements were calculated using a dual-offset approach^29^. To summarize, after the videos are processed, individual frames are removed due to either poor image quality or if the subject blinked. A spatial Gaussian filter was then applied each video frame to smooth the images and reduce noise. Frames were normalized by correcting for overall changes in intensities across all frames and across all pixels throughout the video. To identify vessels, a binary image was generated with white pixels to indicate changes in pixel intensity above an arbitrary threshold based on the temporal variation of the pixels, since temporal variation was highest in the presence of moving blood cells. These thresholded vessels were then segmented, thinned, and labeled in software, allowing vessel segments to be selected by the operator using a mouse.

Figure 4 schematically illustrates the vertical separation of the two beams and their displacement on the retina. The vertical offset between both channels allows for sequential imaging of fine scale details, but with large regions of overlap within the frames, allowing most of the retinal region to be analyzed. After post-processing and analysis via MATLAB software, the two images are realigned automatically.

Once a vessel was selected cells were automatically detected on one of the images based on a z-score metric indicating an object that is present in one frame but not in other frames. The retinal region around detected cells were cropped, generating a region of interest (ROI) of either 12 × 12 or 24 × 24 µm. The same ROI was selected in the second channel. Cropped areas were varied in size by the operator depending on both the size of the vessel and the speed of the RBCs. Depending on the speed, a larger cropped area must be used to detect faster flowing cells. Since the same retinal regions were cropped, but the data were collected with a temporal offset of 3.32 ms in the second channel, cells that are moving will be in different locations in the two ROI’s. To improve the accuracy of measuring the displacement of cells between channels, the displacements were computed by averaging all ROI’s within the selected vessel over three consecutive frames of the video. These spatial averages were oversampled to allow subpixel measurements of cell motion by detecting the centroid of the averaged cells. We then computed the distance between the centroids. Velocity was computed from the displacements and accounting for the impact of the progressive scan speed. That is, because two cells moving at the same velocity downward (with the scan) and upward, will have slightly different displacements, because the scan will encounter them with different delays and thus it is necessary to account for the direction of motion.

We collected 100 frame videos over the entire 2-min stimulus period for each condition, resulting in up to 32 sequences. Usable videos ranged from 5 to 11 for each stimulus condition. Other videos were not used due to the need to have sufficient number of video frames without major eye movements, blinks, or image deterioration between blinks due to dry eye.

### Diameter measurements

Quantification of vessel diameters was performed in Photoshop (Adobe System, Inc., San Jose, CA). To characterize the width of the vessel, which in some cases varies slightly during the cardiac cycle, we selected a high-quality single frame image from each condition. Across all videos acquired for a given condition, we chose a frame based on when the velocity was at its median. Two graders made 5 measurements of the inner diameter of the lumen of each vessel along the selected arteriole and venule. The same approach was used for capillaries unless the wall contrast was too low. In which case we measured the lumen with a motion contrast image of the vessel.

### Blood flow measurements

Once velocity and diameter measurements were made, RBC flow in the vessel can be calculated. Due to the fact that vessels in the microcirculation of the retina exhibit non-Newtonian hemodynamics, a correction factor must be used rather than Poiseuille’s law to account for this^22^. This correction factor assumes that the flow within the smaller vessels are more influenced by shear and other physical interactions than in larger vessels that exhibit laminar flow^56^. This correction factor depends on the average diameter of RBCs and the average diameter of the vessel in which the cells are flowing in. We used the correction factor from studies measuring flow dynamics in small tubes^56,57^. Once the velocity has been corrected, flow can be computed as:

1 $$Q=\frac{{V}_{s}\times \pi \times {D}^{2}}{4}$$

where ${V}_{s}$ is the cross-sectional velocity of RBCs and $Q$ is the total blood flow in the vessel. Total blood flow was computed for each individual’s set of vessels.

### Human subjects

The right eye of nine young healthy subjects (26 years ± 3.8) with no retinal pathology were tested. Imaging sessions with flicker stimulus lasted for approximately 30 min. Prior to imaging, informed consent was obtained from each subject after explanation procedure and goals of the experiment. This research adheres to the tenets of the Declaration of Helsinki and was approved by the Institutional Review Board at Indiana University and all methods were carried out in accords with the guidelines and regulations established. A brief medical history was performed for each subject to ensure no systemic or retinal vascular disease was present. Best-corrected visual acuities were measured, and each subject was dilated with 1% tropicamide. A 30° infrared fundus imaged was obtained and volumetric optical coherence tomography angiography images were obtained, and retinal thicknesses were measured using optical coherence tomography (Spectralis; Heidelberg Engineering, Heidelberg, Germany). Axial lengths of each eye were also measured (IOL Master, Zeiss Instruments).

### AO imaging sessions

**Figure 5:**
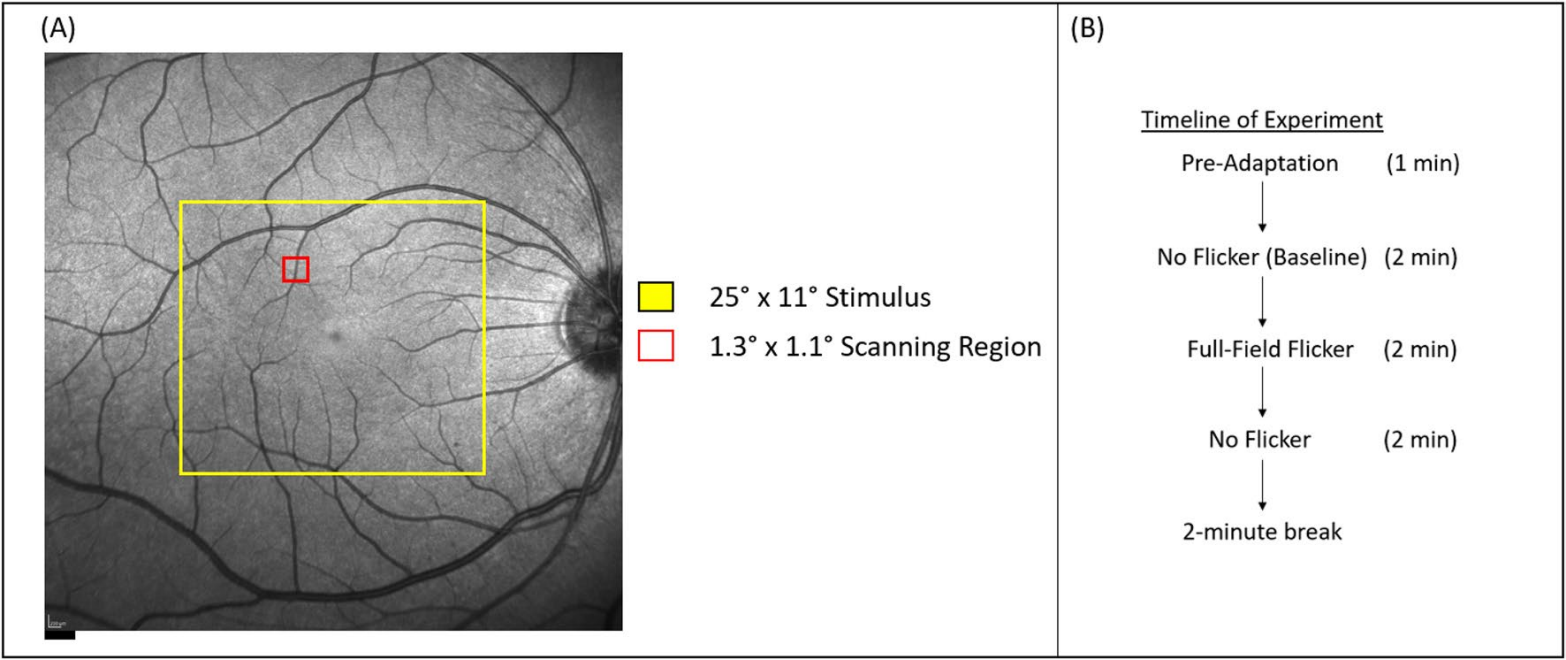
(**A**) Fundus image of one subject showing the location of both the raster scan and the flicker stimulation. (**B**) A timeline establishing the duration of the Full-Field Flicker experiment. Scale bar is 500 µm.

The AO imaging sessions was divided into two sessions: The first session included a protocol to acquire cone and vascular images of the macula by imaging in both a 2.0° × 1.75° imaging field encompassing 6° × 6° around the macula, and a 1.3° × 1.1° imaging field in the same locations, a 14° × 3° strip-wise region of cones and vasculature temporally from the fovea, and a 14° × 3° strip-wise imaging region of the vasculature of the superior retina (beginning 2° superior from the fovea). From the vascular images collected at the superior retina, an interconnected vascular region in the superior macula was selected for the second AO session involving the flicker stimulation. Each interconnected region selected included an arteriole, capillaries, and venule. Custom MATLAB software was then used the subject’s fundus image and the scanner positions to sample the retina at the desired locations. The criterion for selecting arterioles and venules was based on both the size of the vessels as well as the ability to visualize multiple capillaries in a region between both vessels. Arterioles and venules were identified by comparing AOSLO montages to 30 degree SLO images, as well as confirming that the direction of flow measured with the AOSLO was consistent with the vessel type. A small imaging field (1.3° × 1.1°) was used for identifying RBCs. For the second AO session, subjects were imaged in three sets of three-2-min imaging sessions consisting of (1) a baseline no flicker condition, (2) the full-field flicker condition, and then (3) a no flicker condition. This third condition allowed us to measure a return to baseline velocities. Prior to measuring the baseline velocities, subjects sat in the system for 1 min while fixating on the uniform background to pre-adapt the retina to the average illuminance, helping to ensure a stable baseline. The order of vessel measurements was randomized prior to the beginning of the imaging session. Before imaging the next vessel location, subjects were given a 2-min break. During each break, artificial tears were instilled to ensure adequate corneal tear quality. The computer used to generate the visual stimuli was also used to control and record experimental timing. Figure 5 illustrates the area of a vessel region in one subject relative to the size of the flicker stimulus (A) as well as the timeline for each flicker condition (B).

### Statistical analysis

Statistical analysis was performed using Microsoft Excel software (Microsoft, Redmond, WA) with a significance level set at p < 0.05. Axial velocity, diameter, and flow were normalized by comparing the average velocity, diameter, and flow in condition 2 (full-field flicker) and condition 3 (after flicker stimulation) relative to condition 1 (pre-flicker baseline) in each subject. To measure the effect of flicker stimulation on each vessel, a single-factor ANOVA was used to compare the normalized velocities, diameter, and flow across each condition. To determine whether the full-field flicker stimulus condition was the determinant factor for increases in hemodynamic response, a Bonferroni post-hoc t-test was performed to compare differences in condition 2 and condition 3 relative to baseline. Lastly, the coefficient of variation (C.V.) was used to measure fluctuations in RBC axial velocity in each type of vessel for each experimental condition. The C.V. was computed by dividing the standard deviation of the average velocities by the mean over all subjects.

## Supplementary information

Supplementary Figures.
