# Supplementary material for: Full-field flicker evoked changes in parafoveal retinal blood flow
Source: Sci Rep. 2020 Sep 29;10:16051. doi: 10.1038/s41598-020-73032-0 (PMC7524838; doi:10.1038/s41598-020-73032-0)
Supplement: Supplementary file 1 — Supplementary Figures. [file 41598_2020_73032_MOESM1_ESM.docx]

**Full-Field Flicker Evoked Changes in Parafoveal Retinal Blood Flow**

Raymond L. Warner^1,*^, Alberto de Castro^1^, Lucie Sawides^1^, Tom Gast^1^, Kaitlyn Sapoznik^1^, Ting Luo^1^, Stephen A. Burns^1^

^1^Indiana University, School of Optometry, Bloomington, 47405, United States of America

*Corresponding Author: [raylwarn@iu.edu](mailto:raylwarn@iu.edu)

**Supplementary Figures**


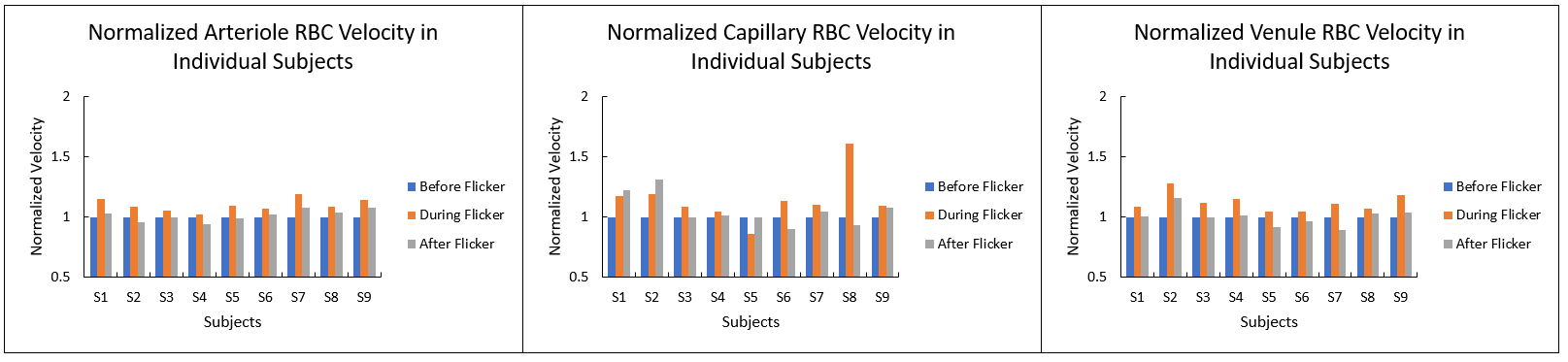


Supplementary Figure 1. Velocity measurements for each vessel type in individual subjects for each condition. Velocities were normalized by dividing the average velocity for each condition to the baseline velocity measurement.


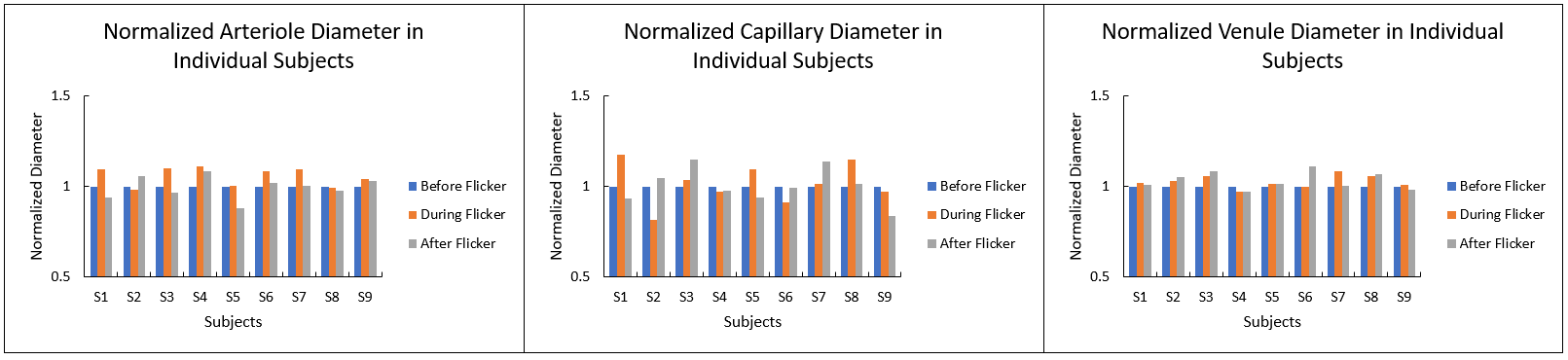


Supplementary Figure 2. Diameter measurements for each vessel type for each subject for each condition. Diameters were normalized by dividing the average velocity for each condition to the baseline velocity measurement.


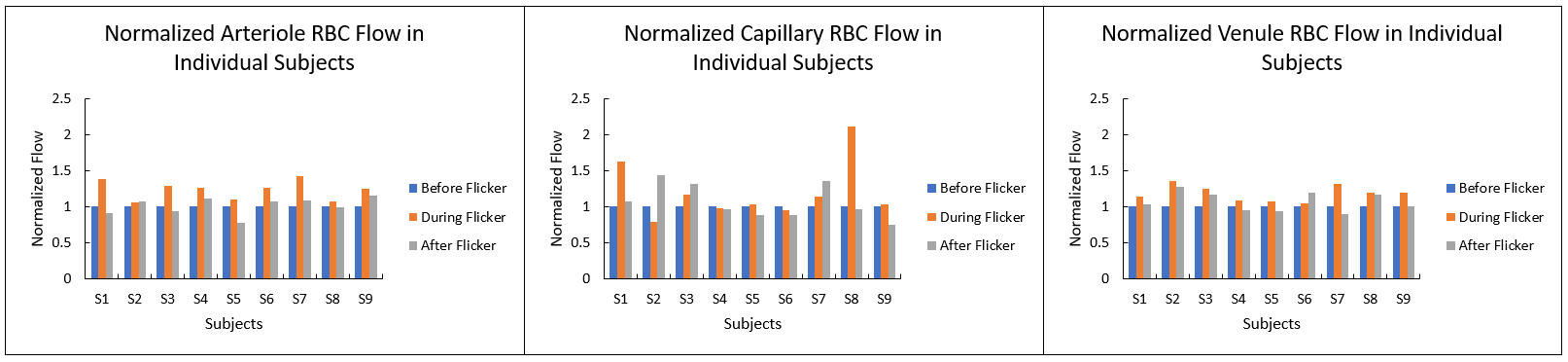


Supplementary Figure 3. Individual flow measurements for each vessel type during each condition. Flow measurements were normalized by dividing the average velocity for each condition to the baseline velocity measurement.


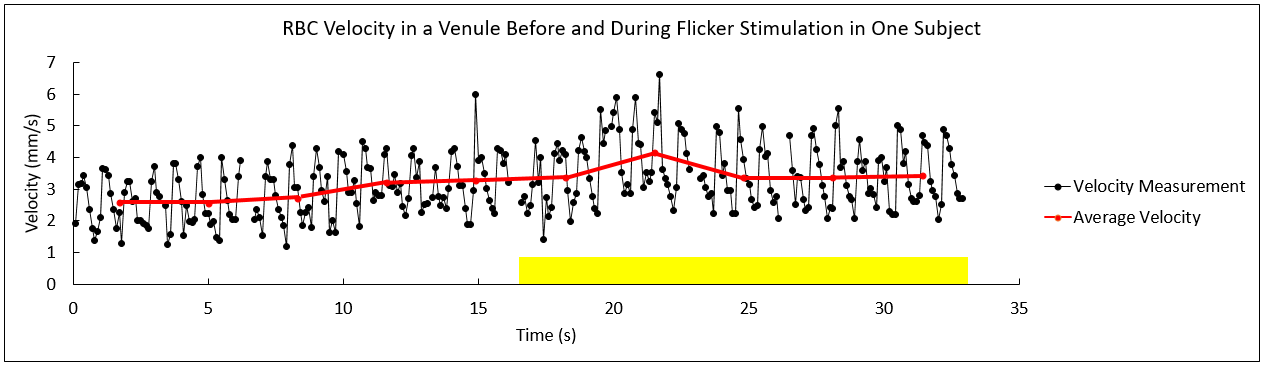


Supplementary Figure 4. 10 measurements of the axial velocity of a subject’s RBC velocity in a venule. The first 5 measurements (from 0.0 seconds-16.3 seconds) are taken from the last 5 videos recorded during condition 1 (No Flicker). The next 5 measurements (from 16.6 seconds-33.0 seconds) are the first 5 measurements taken at the beginning of condition 2 (Full-Field Flicker Stimulation). The yellow bar represents the time course of the flicker stimulus. During onset of flicker stimulation, we can observe changes in the average velocity in an individual vessel as it reaches peak velocity about 5 seconds after flicker stimulation, then stabilizes its average velocity.
